# Investigating Functioning Profile of Adolescents with Anorexia before and during the COVID-19 Pandemic: A Cross-Sectional Study on Mentalizing, Alexithymia, and Impulsiveness

**DOI:** 10.3390/ijerph20043670

**Published:** 2023-02-19

**Authors:** Fabiola Bizzi, Anna Riva, Simone Charpentier Mora, Marta Tironi, Sofia Elena Sforza, Lorenzo Maria Milani, Renata Nacinovich

**Affiliations:** 1 Department of Educational Sciences, University of Genoa, Corso A. Podestà 2, 16121 Genoa, Italy; 2 Child and Adolescent Mental Health Department, Fondazione IRCCS San Gerardo dei Tintori, University of Milano-Bicocca, Via Pergolesi 33, 20900 Monza, Italy; 3 School of Medicine and Surgery, University of Brescia, 25123 Brescia, Italy; 4 NeuroMI—Milan Center for Neuroscience, University of Milano-Bicocca, 20126 Milan, Italy

**Keywords:** anorexia nervosa, psychological difficulties, mentalizing, alexithymia, impulsiveness, adolescence, COVID-19 pandemic

## Abstract

Anorexia nervosa (AN) usually emerges in adolescence when important changes occur in cognitive, emotional, and social processes. Mentalizing, alexithymia, and impulsiveness represent key dimensions for the understanding and interpretation of psychological difficulties in AN. The outbreak of the COVID-19 pandemic has impacted adolescents with AN, showing a worsening of the disease. The main aims of the present paper are (1) to compare adolescents with AN before and during the COVID-19 pandemic and (2) to explore the relationship between mentalizing, alexithymia, impulsiveness, and psychological difficulties related to eating disorders in adolescents with AN during the COVID-19 pandemic. One hundred and ninety-six AN female adolescents (N = 94 before COVID-19; N = 102 during COVID-19) participated in this study. The results show that adolescents with AN during the COVID-19 pandemic had a more impaired functioning profile than the before COVID-19 group. Mentalizing, alexithymia, and impulsiveness had a predictive role on psychological difficulties related to eating disorders in adolescents with AN during the COVID-19 pandemic. In conclusion, our data reveal that the COVID-19 pandemic has likely represented a stress condition that affects mental health; worsening the severity of adolescents with AN clinical condition. Lastly, predictive patterns suggest the existence of a link between difficulties in the ability to face the problems of the present time using effective strategies and the severity of psychological symptoms.

## 1. Introduction

Anorexia nervosa (AN) is a serious eating disorder, which is more often diagnosed in females and usually has an onset in adolescence. It is characterized by impaired body experience and perception, leading to self-starvation and an underweight condition linked to an intense fear of gaining weight, and difficulty in emotional management. It may be classified as restrictive or binge eating/purging subtypes depending on the presence of behaviors such as binge eating, purging, or other compensatory strategies such as excessive exercising or fasting [1].

In recent years, the constructs of mentalizing, operationalized as reflective functioning (RF) (i.e., the capacity to reflect on and interpret one’s behavior and that of others based on intentional internal mental states, such as beliefs, thoughts, and emotions [2]), and alexithymia (i.e., the inability to verbally describe and identify moods, as well as restricted imaginative capacities [3]) have attracted increasing research interest in the field of AN, representing aspects of social and emotional development salient to assess and treat this disorders [4]. Overall, individuals become vulnerable to interpersonal factors when mentalizing—this develops within the social matrix of attachment relationships which are impaired—the individuals lack a secure bond with a caregiver that allows the development of a coherent self-structure [2]. Similarly, deficiency of regulation and recognition of emotions puts the adaptive development of the individual at serious risk predisposing it to psychological difficulties and mental problems including AN [5].

According to the literature [6,7,8,9], patients with AN exhibited significantly lower levels of mentalizing compared with community controls. Rothschild-Yakar et al. [10] found that a higher RF was associated with a lower drive for thinness in their community sample. However, in their clinical sample, which consisted of inpatients with AN binge/purge subtypes, no correlation was found between mentalizing and the drive for thinness. In another study, a positive correlation was found between mentalization and bulimic symptomatology, pointing out that bulimic patients were more polarized in their RF abilities, with higher scores in both the low and high ranges [11].

In addition, a developed ability to mentalize is considered a key factor in the development of adequate affect-regulation abilities [2] and along these lines, patients with AN have elevated levels of alexithymia compared to patients with bulimia nervosa (BN) and controls [5,12,13]. This suggested that anorexic behaviors may partly help to avoid possible negative sensations and the difficulties of emotion regulation [14,15]. Conversely, impulsiveness (i.e., the incapacity related to the essential control of thoughts and behavior; [16]), in patients with AN being mainly linked with affective instability typical of bulimic rather than anorexic symptomatology, where self-control, caution, and conscientiousness prevail, has rarely been investigated. Despite recent studies indicating impulsiveness—intended as impulsive behavior, such as fast inaccurate responses—is associated with nonsuicidal self-injury (NSSI) typical of severe forms of AN [17,18], this remains an overlooked aspect in AN research. This led us to refer to mentalizing and affect regulation as processes that are closely interrelated in patients with AN [10]. However, they are rarely investigated in adolescence when the changes a person faces to find a place in society and among his or her peers, and the modification of his/her body induced by puberty, can lead to emotional difficulties [12]. In addition, the relationship between individual differences in mentalizing, alexithymia, impulsiveness, and psychological difficulties in adolescence is poorly understood. One of the hallmarks of adolescence is socio–emotional vulnerability, accompanied by a heightened risk for the emergence or exacerbation of psychopathological symptoms. It is possible to hypothesize that psychological difficulties, considered on a continuum from typical to pathological manifestations, could have a particularly important impact on social cognition schemas, and notably, on developing these skills [12,18,19].

As mentioned, the number of studies that have examined mentalizing in adolescents with AN is relatively small, reporting controversial results due to a variety of different methods used to assess the different facets of the disease investigated and a relatively poor validity of the instruments themselves [4,20,21]. As a matter of fact, a higher level of alexithymia in adolescents with AN is easily found using the Toronto alexithymia scale (TAS-20) as a gold-standard measure showing a link between body symptoms and difficulties in managing and expressing emotions at the symbolic verbal level [15,22,23]. Nevertheless, mentalizing still needs to be further investigated in adolescents with AN, with well-validated measurements considering the existence of different mentalizing polarities and varying between diagnoses of different eating disorders [24,25]. Similarly, impulsiveness needs to be further investigated with well-validated measurements considering the existence of different impulsiveness polarities not yet studied in adolescents with AN [17]. 

The outbreak of the Coronavirus disease 2019 (COVID-19) pandemic with the consequent adoption of social and physical distancing measures to contain virus transmission has impacted patients with AN, in terms of showing a worsening psychopathology and heightened vulnerability in terms of physical distress and psychological difficulties [26]. The literature shows a heightened food restriction, increased excessive physical exercise, more frequent symptoms and worries, a decrease in social support, and increased exposure to video conferencing and social media related to thin-ideal and diet culture [27]. These data are corroborated by the observed increase in the number of urgent and routine referrals of individuals with eating disorders (EDs) during the pandemic, as well as by the increase in patient admissions, especially of adolescents with EDs [28]. Consistently, the COVID-19 pandemic may represent a traumatic stress condition that affected subjective mental health by increasing perceived insecurity, undermining the mentalizing abilities when the attachment system is stimulated, and reducing the range of strategies through which people usually regulate their emotions [29,30,31].

Given the large impact COVID-19 has on mental health, the aims of the current study were:

(a) To compare adolescents with AN with respect to mentalizing, alexithymia, and psychological difficulties related to eating disorders before and during the COVID-19 pandemic;
(b) To investigate the relationship between mentalizing, alexithymia, impulsiveness, and psychological difficulties related to eating disorders in adolescents with AN during the COVID-19 pandemic.

For the first objective, it is hypothesized that adolescents with AN diagnosed during the COVID-19 pandemic will show a deficit in mentalizing, higher levels of alexithymia, and more psychological difficulties related to eating disorders compared to adolescents with AN diagnosed before the COVID-19 pandemic. For the second objective, the hypothesis is that a deficit in mentalizing, alexithymia, and impulsiveness will be associated with psychological difficulties related to eating disorders in adolescents with AN during the COVID-19 pandemic. 

## 2. Materials and Methods

### 2.1. Research Design and Participants

The research design consisted of a case–control (i.e., a study that compares two groups of people—AN patients diagnosed during the pandemic as cases and a very similar group of AN patients diagnosed before the pandemic as controls) and cross-sectional study. Data were collected from medical records in the last two years before (from September 2018 to February 2020) and during (from August 2020 to May 2022) the pandemic. Considering the low percentage (N = 13) of males with AN admitted to our center in the whole period, we decided to exclude males from our research, to get a more homogeneous sample. One hundred and ninety-six Italian female adolescents (aged 11–17 years; mean age = 14.94, SD = 1.60) were enrolled in this study. They were recruited as patients at the Child and Adolescent Mental Health Department, ASST Monza, University of Milano-Bicocca (Monza, Italy) for severe malnutrition and diagnosed with anorexia nervosa (AN) according to DSM-5 criteria [1]. 

The participants were divided into two groups: a group (before COVID AN) recruited before the pandemic and composed of 94 female adolescents, and a group (During-COVID AN) recruited during the COVID-19 pandemic and composed of 102 female adolescents. The socio–demographic characteristics of the two groups are reported in Table 1.

The inclusion criteria for both samples were adolescent age and diagnosis of AN. The exclusion criteria were: the presence of psychotic disorders, other eating disorders, intellectual disabilities, and neurological disorders. The presence of personality disorders in comorbidity has not been evaluated since the mean disease duration of the sample is less than 1 year, a time too short to diagnose a personality disorder according to DSM-5 [1].

### 2.2. Variables and Measurements 

An ad hoc socio–demographic questionnaire was used to collect detailed information about the participants, including gender, age, and medical history information (i.e., body mass index (BMI), nonsuicidal self-injury (NSSI), and suicidal ideation).

#### 2.2.1. Symptomatology Associated with Eating Disorders

The Eating Disorder Inventory-3 (EDI-3) [32] is a self-report instrument designed to evaluate eating disorder pathology. It consists of 91 items organized into 12 primary scales, 3 specific eating disorder scales, and 9 general psychological scales that are highly relevant, but not specific, to eating disorders. It also yields six composite scores, one which is eating-disorder specific (eating disorder risk, EDRC) (i.e., “I am terrified of gaining weight” or “I have gone on eating binges where I have felt that I could not stop”) and five which tap into general integrative psychological constructs (ineffectiveness, IC; interpersonal problems, IPC; affective problems, APC; overcontrol, OC; and global psychological maladjustment, GPMC) (i.e., “I get confused about what emotion I am feeling” or “I feel inadequate”). The participants respond to the items on a 6-point Likert scale, and for the scoring system they are recoded as 0, 1, 2, 3, and 4. In this study, we used the Italian version [33] which shows good psychometric properties for adolescents and adults, and we considered the results of the six composite scales only. The reliability coefficients of the scales ranged from 0.80 to 0.90, and test–retest reliability coefficients for the various composite scales were between 0.93 and 0.98.

#### 2.2.2. Mentalizing

The reflective functioning questionnaire (RFQ) [34] is a 7-point Likert-type self-report 8-item questionnaire used to evaluate mentalizing abilities by measuring the degree of certainty (RFQc subscale) and uncertainty (RFQu subscale) with which individuals utilize mental state information to understand their own and others’ behavior. The RFQc subscale focuses on the extent to which individuals disagree with statements such as “I don’t always know why I do what I do”). Very low agreements on this scale reflect hypermentalizing, while some agreement reflects adaptive levels of certainty about mental states. The RFQu subscale is composed of items such as “Sometimes I do things without really knowing why”. High scores reflect a stance characterized by an almost complete lack of knowledge about mental states, while lower scores reflect an acknowledgment of the opaqueness of one’s mental states and those of others, typical of genuine mentalizing. The Italian adaptation version for adolescents with 6 items used in this research [35] shows good internal consistency in both subscales (Cronbach’s α higher than 0.70).

#### 2.2.3. Alexithymia

The Toronto alexithymia scale (TAS-20) [36], is a 5-point Likert-type self-report 20-item questionnaire used to assess the alexithymia in the total level (TAS–Total) and in the three aforementioned factors: difficulties in identifying feelings (TAS–DIF, e.g., “I am often confused about what emotion I am feeling”), difficulties in describing feelings (TAS–DDF, e.g., “I find it hard to describe how I feel about people”), and lack of focus on internal emotional experiences (TAS–EOT, e.g., “I prefer to analyze problems rather than just describe them”). Scores < 51 suggest no alexithymia, 51–60 border-alexithymia and >61 alexithymia. The Italian version used in this research [37] shows good internal consistency in all subscales (in this study Cronbach’s α higher than 0.72).

#### 2.2.4. Impulsiveness

The Barratt Impulsiveness Scale-11 (BIS-11; [16]) is a 4-point Likert-type self-report 30-item questionnaire used to assess different aspects of impulsiveness. The items are divided into three subscales: attentional impulsiveness (cognitive instability and inattention; BIS–Attentional, e.g., “I do not pay attention”), motor impulsiveness (acting on the spur of the moment and lack of perseverance; BIS–Motor, e.g., “I act on the spure of the moment”), and non-planning impulsiveness (intolerance of cognitive complexity and lack of self-control; BIS–Planning, e.g., “I am a careful thinker”), and a total score. The total score ranges from 30 to 120, with a higher score indicating more impulsiveness. The Italian version used in this research [38] shows good internal consistency of all subscales (in this study Cronbach’s α higher than 0.79).

### 2.3. Procedure

The procedure was approved by the research ethical committee of ASST Monza, and complied with the ethical standards of the international scientific community. All the parents provided written and informed consent before the assessment was administered. Participants were assessed in the hospital in an individual session by a clinical researcher.

### 2.4. Statistical Analysis

Data were analyzed using Statistical Package for the Social Sciences (SPSS, version 24; IBM Corp., Armonk, NY, USA). Preliminary tests of skewness and kurtosis were used to determine whether data of each scale and subscale were normally distributed, showing no violation of normality. Demographic variables were described using descriptive statistics (frequencies and percentages for the categorical variables and means and standard deviations for the continuous variables). At the beginning, we tested if confounding/socio–demographic variables were different between groups.

To test the first hypothesis, Chi-squares were used to compare two groups on categorical variables and t-tests or ANOVAs for comparisons of continuous variables. Comparisons for mentalizing, alexithymia, and psychological difficulties related to eating disorders in the two groups were conducted through multivariate analyses (ANCOVA, MANCOVA) using age, BMI, NSSI, and type of AN as covariates. To explore the second hypothesis, Pearson’s correlations were computed only in the COVID-19 group in order to study the association between mentalizing, alexithymia, impulsiveness, and psychological difficulties related to eating disorders, while controlling the effect of age, BMI, NSSI, and type of AN. Finally, significant correlations were included in regression analyses with RFQ, TAS-20, and BIS-11 as independent variables and EDI-3 as dependent variable. A *p*-value < 0.05 was considered significant for all analyses. There was no evidence of multicollinearity problems (tolerance values > 0.05 and VIF values < 2 for all models).

## 3. Results

### 3.1. Mentalizing, Alexithymia, and Psychological Difficulties Related to Eating Disorders before and during the COVID-19 Pandemic

#### 3.1.1. Preliminary Analyses and Descriptive Statistics

Preliminary analyses were conducted in order to explore a link between the study variables and confounding/socio–demographic and medical history information variables (i.e., age, BMI, NSSI, and type of AN). Firstly, correlation analyses in the whole sample showed a significant relationship between age and (1) all composite EDI scales (all *p* < 0.05), except for GPMC; (2) RFQc (*p* < 0.001); and (3) TAS–Total and TAS–DDF (both *p* < 0.01). Moreover, there was a significant relationship between BMI and (1) all composite EDI scales (all *p* < 0.05), except for IPC; (2) RFQu (*p* < 0.001); and (3) all TAS scales (TAS–Total, TAS–DIF, TAS–DDF; all *p* < 0.03), except for TAS–EOT. Secondly, we conducted 2X3 MANOVAs, including NSSI at admission and type of AN as independent variables, and EDI composite scales, TAS scales, and RFQ scales as dependent variables. Analysis showed a significant relationship between NSSI and (1) all composite EDI scales (all *p* < 0.001), except for EDRC and OC; (2) all TAS scales (TAS–Total, TAS–DIF, TAS–DDF; all *p* < 0.05), except for TAS–EOT; and (3) RFQu (*p* = 0.009). Even type of AN showed relationship with some composite EDI scales (EDRC, IC, APC, OC, all *p* < 0.02).

In conclusion, age, BMI, NSSI, and type of AN were included as covariates in subsequent analyses when necessary.

#### 3.1.2. Comparison between Groups before and during the COVID-19 Pandemic

In order to explore differences in mentalizing, alexithymia, and psychological difficulties related to eating disorders before and during the COVID-19 pandemic, we initially performed some analyses to verify if age, BMI, NSSI at the time of admission, and type of AN differed between groups but there were no differences (see Table 1). Next, we compared the two groups based on results in RFQ, TAS-20, and EDI-3 taking into account the above-mentioned covariates (see Table 2 for means and standard deviations).

Regarding mentalizing, we found statistically significant differences between groups only in uncertainty about mental states (RFQu; F (1, 164) = 5.25, *p* = 0.023), but not in certainty about mental states (RFQc; F (1, 168) = 1.88, *p* = 0.99). Patients assessed during the COVID-19 pandemic showed a higher level of uncertainty in mentalizing than before-COVID-19 pandemic patients with AN.

Concerning alexithymia, in TAS–Total (F (1, 156) = 5.60, *p* = 0.019) and in TAS–DDF (F (1, 156) = 6.42, *p* = 0.012) there were statistically significant differences with the group assessed during the COVID-19 pandemic showing higher scores than the before-COVID-19 group. TAS–EOT (F (1, 163) = 0.45, *p* = 0.50) and TAS–DIF (F (1, 158) = 3.53, *p* = 0.06), did not show any differences.

Lastly, regarding psychological difficulties related to eating disorders, the analyses revealed that the eating disorder risk score (EDRC; F (1, 141) = 18.46, *p* < 0.001), and nearly all the general integrative psychological constructs (ineffectiveness, IC; interpersonal problems, IPC; affective problems, AP; overcontrol, OC) were significantly higher in the group assessed during COVID-19 (with all *p* < 0.05) except for the general psychological maladjustment scale (GPMC; *p* = 0.262) which did not differ between the groups.

Therefore, our first hypothesis was supported: controlling for age, BMI, NSSI, and type of AN, patients assessed during COVID-19 showed a more impaired functioning profile in mentalizing, alexithymia, and worse psychological characteristics than the before-COVID-19 group.

### 3.2. Relation between Mentalizing, Alexithymia, and Impulsiveness on Psychological Difficulties Related to Eating Disorders during the COVID-19 Pandemic

#### 3.2.1. Preliminary Analyses and Descriptive Statistics

In order to test the predictive role of mentalizing, alexithymia, and impulsiveness on psychological difficulties related to eating disorders in AN adolescent patients, we used the EDI composite scales (i.e., EDRC, IC, IPC, AP, OC, GPMC) as dependent variables. Having checked for confounding variables on these scales, age (OC and GPMC *p* < 0.05), BMI (APC, OC, and GPMD all *p* < 0.05), NSSI (IC, APC, GPMC all *p* < 0.05), and suicidal ideation (APC *p* < 0.05) revealed significant relationships and thus were included in subsequent analyses as covariates.

#### 3.2.2. Effect of Mentalizing, Alexithymia, and Impulsiveness on Psychological Difficulties Related to Eating Disorders

In order to examine the relationship between specific dimensions of mentalizing (RFQc and RFQu), alexithymia (TAS–DIF, TAS–DDF, TAS–EOT), and impulsiveness (BIS–Attentional, BIS–Planning, and BIS–Motor), and EDI composite scales, first we ran correlation analyses. Significant correlations are reported in Table 3, while RFQc, BIS–Planning, and BIS–Motor showed no significance.

We then used a series of linear regression models that proved to be statistically significant. Table 4 reports the main parameters of regression model 1 with EDI–EDRC as the dependent variable and BIS–Attentional, TAS–DDF, and TAS–EOT as predictors. Overall, the final model is statistically significant and explains about 12% of the variability in the risk to develop an eating disorder (F (3, 55) = 3.72, *p* = 0.02): the cognitive instability and inattention (BIS–Attentional; b = 0.33, SE = 0.66, *p* = 0.011) is a statistically significant predictor of the risk to develop an eating disorder. Once the TAS–DDF (b = 0.16, SE = 0.65, *p* = 0.26) and TAS–EOT (b = 0.17, SE = 0.52, *p* = 21) scales were included, however, the BIS–Attentional scale lost its significance (b = 0.24, SE = 0.70, *p* = 0.079). In conclusion, no scale has a specific effect on the risk of developing eating symptoms.

Table 5 reports the main parameters of regression model 2 with EDI–GPMC as the dependent variable and RFQu, BIS–Attentional, TAS–DIF, and TAS–DDF as predictors. Overall, the final model was statistically significant explaining about 64% of the variability in developing general psychological maladjustment (F (7, 44) = 13.74, *p* < 0.001). Specifically, NSSI (b = −0.62, SE = 5.44, *p* = 0.03), the BIS–Attentional scale (b = 0.49, SE = 0.60, *p* < 0.001), RFQu (b = 0.24, SE = 3.34, *p* = 0.04), TAS–DIF (b = 0.39, SE = 0.33, *p* < 0.01), and TAS–DDF (b = 0.28, SE = 0.48, *p* = 0.02) were significant predictors. In the final model, both NSSI and RFQu became non-significant.

Table 6 reports the main parameters of regression model 3 with EDI–IC as the dependent variable and RFQu, BIS–Attentional, TAS–DIF, and TAS–DDF as predictors. Overall, the final model was statistically significant explaining about 48% of the variability in developing a sense of Ineffectiveness (F (5, 48) = 11.53, *p* < 0.001). Specifically, NSSI (b = −0.75, SE = 5.42, *p* = 0.005), the BIS–Attentional scale (b = 0.40, SE = 0.67, *p* = 0.001) and TAS–DDF (b = 0.40, SE = 0.57, *p* = 0.002) were significant predictors. In the final model, both NSSI and BIS–Attentional became non-significant.

Table 7 reports the main parameters of regression model 4 with EDI–IPC as the dependent variable and BIS–Attentional, TAS–DIF, and TAS–DDF as predictors. Overall, the final model was statistically significant explaining about 47% of the variability in developing interpersonal problems (F (3, 55) = 18.33, *p* < 0.001). Specifically, the BIS–Attentional scale (b = 0.44, SE = 0.79, *p* < 0.001) and TAS–DDF (b = 0.62, SE = 0.72, *p* < 0.001) were significant predictors. In the final model, BIS–Attentional became marginally significant.

Table 8 reports the main parameters of regression model 5 with EDI–APC as the dependent variable and RFQu, BIS–Attentional, TAS–DIF, and TAS–DDF as predictors. Overall, the final model was statistically significant explaining about 65% of the variability in affective problems (F (7, 50) = 16.37, *p* < 0.001). Specifically, NSSI (b = -.88, SE = 5.69, *p* = 0.001), the BIS–Attentional scale (b = 0.43, SE = 0.57, *p* < 0.001) and TAS–DIF scale (b = 0.63, SE = 0.36, *p* < 0.001) were significant predictors. In the final model, NSSI became non-significant.

Table 9 reports the main parameters of regression model 6 with EDI–OC as the dependent variable and BIS–Attentional, TAS–DIF, and TAS–DDF as predictors. Overall, the final model was statistically significant explaining about 28% of the variability in showing overcontrol (F (5, 49) = 5.15, *p* < 0.001). Specifically, age (b = 0.33, SE = 2.27, *p* = 0.01), the BIS–Attentional (b = 0.36, SE = 0.89, *p* = 0.005) and TAS–DIF (b = 0.36, SE = 0.63, *p* = 0.05) were significant predictors. In the final model, the BIS–Attentional became marginally significant.

## 4. Discussion

In this study AN female adolescents were assessed in mentalizing, alexithymia, and impulsiveness, aiming at identifying pathways of risk connected to psychological difficulties related to eating disorders and comparing the psychological functioning of these patients before and during the COVID-19 pandemic.

Starting from the first hypothesis, the results confirmed that patients with AN assessed during the COVID-19 showed more impaired mentalizing, higher levels of alexithymia, and greater psychological difficulties than those assessed during the beforeCOVID-19 pandemic period. This suggests that, as hypothesized, the COVID-19 pandemic represents a stressful condition which affects subjective mental health undermining mentalizing abilities and reduces the range of strategies through which people usually regulate their emotions [29,30,31]. Specifically, adolescents with AN during this pandemic hardly utilized mental state information to understand their own and others’ behavior. They were overwhelmed by heightened emotions, difficult to identify and describe, and showed more interpersonal and affective problems than adolescents with AN before the COVID-19 pandemic. In that sense, mentalizing difficulties, alexithymia, and impulsiveness became more pronounced during the pandemic, making adolescents more vulnerable to developing psychological difficulties related to eating disorders [28]. These aspects could be particularly important for adolescents who are experiencing this specific transitional period characterized by profound changes in the development of the self, and could affect the perturbing period represented by the spread of the COVID-19 pandemic [39], determining a complex interplay between developmental tasks and environmental conditions that may increase the risk for the onset of psychological difficulties related to eating disorders or the severity of these clinical conditions. In addition, isolation and forced cohabitation may have led to restrictions in personal freedom, which caused tensions between patients and family members, exacerbating their psychological distress. This may have affected all those abilities, such as emotional regulation and mentalizing, associated with attachment bonds, strongly linked to the development of eating disorders in adolescence [4].

As to the second hypothesis, the results confirm that mentalizing difficulties, alexithymia, and impulsiveness are associated with psychological difficulties related to eating disorders in adolescents with AN during the COVID-19 pandemic. In particular, some specific polarities of alexithymia, such as describing and identifying feelings, and impulsiveness as indicator for cognitive instability and inattention were associated with a sense of ineffectiveness, interpersonal and affective problems, and overcontrol of adolescents with AN. In addition, the uncertainty with which adolescents with AN utilize mental state information to understand their own and others’ behavior was associated with a sense of ineffectiveness and affective problems. This complex pattern might suggest the presence of difficulties in the ability to face the problems of the present time using emotion regulation strategies that take into account one’s and others’ mental states by allowing a sense of self-efficacy. In line with some recent studies, patients with AN would show a disorder related to the affective components of experiencing another person’s experience, while retaining, instead, certain more intellectual abilities to recognize and understand it [39]. Additionally, not surprisingly, studies suggest a high incidence of attachment difficulties in patients with AN which are also associated with difficulties in emotion regulation, and they are both connected with eating problems [14,40,41]. All of our variables are associated with patients with AN, global psychological maladjustment, and eating disorder risk in this direction. On the contrary, the certainty of mental state, the lack of focus on internal emotional experiences, the intolerance of cognitive complexity, the motor impulsiveness by lacking perseverance, and the lack of self-control are not associated with psychological difficulties related to eating disorders, suggesting that these specific polarities of mentalizing, alexithymia, and impulsiveness are distinct non-prototypical processes of adolescents with AN. This is not surprising considering the central role performed by specific polarities of the variables investigated in influencing reported psychological symptoms. The two dimensions of RF for example displayed different functions of mentalizing suggesting that only uncertainty about mental states may be a good marker of typical features associated with clinical problems [33]. Moreover, regression analyses, checking for confounding variables such as age, BMI, NSSI, and suicidal ideation, showed that alexithymia dimensions were the stronger predictors of psychological difficulties related to eating disorders differently from mentalizing and impulsiveness. This suggests that this factor limits the development of the self and the interpersonal relationship, and that it is closely related to deficits in the perception of both one’s and others’ feelings. It is also associated with the type of patients with maladaptive defense mechanisms, including image-distorting style defense mechanisms and self-sacrificing style defense mechanisms [42]. A recent review showed that even after specific treatment, alexithymia levels can persist at high levels [43]. Furthermore, impairment in emotional regulation and awareness are evident in adult AN patients [44]. So, in clinical routine it will be crucial to assess these variables and plan tailored interventions starting from adolescence. Our findings are in line with research in psychotherapy on AN patients that pointed out how mentalization-based treatment (MBT) could be useful to rehabilitate these functions with a focus on how the body represents mental states [44]). According to preliminary studies, MBT may be effective in treating eating disorders and their co-morbid nonsuicidal self-injury symptoms [45,46].

Limitations to this study include the enrollment of females only and the use of self-report questionnaires to assess psychological variables. Recruiting male subjects and more rigorous approaches to the measurement, for instance by combining self-report instruments with interview measures or also adopting objective measurements for impulsiveness would be important [4,17]. Conversely, the strengths of this study include a large and homogeneous sample of adolescents exclusively with anorexia nervosa, improving the quality of the results and reducing possible biases. Moreover, we investigated internal variables in adolescence, a developmental phase which is of increasing clinical interest, and the associations among the above-mentioned variables in a very unusual period, namely the COVID-19 pandemic, fraught with spillover effects on youths’ mental health, as largely evidenced by research in recent years [47]. Further research should be conducted, evaluating the co-presence of personality disorders, and considering other subtypes of eating disorders in adolescence. Other important perspectives for future research include longitudinal studies, to evaluate the construct of mentalizing, alexithymia, and impulsiveness during illness, and the evolution of illness in relation to the presence of these processes.

## 5. Conclusions

In conclusion, our research confirmed the hypothesis according to which mentalizing difficulties, alexithymia, and impulsiveness are risk factors linked to AN in adolescents who have worsened during the COVID-19 pandemic. Given the large impact that AN has on mental health, societal costs, and quality of life, these findings indicate that under conditions of stress, adolescents’ social understanding and social functioning need to be supported by tailored psychological programs. The worsening results obtained from the comparison of before- and during-the-pandemic situations, emphasizes once again the importance of emotional–relational fallout from the home environment as a possible risk factor for the development of AN, confirming the need to provide family interventions that are effective, efficient, and cost-effective [48]. At the individual level, knowledge of how patients understand the relationships between their emotions and their anorexic behavior can be an important addition to treatment programs for AN. However, this may not be enough. In this sense, a therapist’s mentalizing stance that facilitates the opening up of the mind to learning from the social context of the young patient, and to developing the belief that it is possible to acquire relational knowledge in therapy, may be equally crucial in dealing with adolescents with AN [49].

## Figures and Tables

**Table 1:** Socio–demographic characteristics of the sample and comparison between groups.

	Before-COVID-19 Group (n = 94)	During-COVID-19 Group (n = 102)	Comparison between Groups
	Mean (SD)	Mean (SD)	Statistics
Age	14.8 (1.55)	14.9 (1.64)	*t* = −0.45, *p* = 0.652
Body mass index (BMI) at admission	16.2 (2.66)	16.2 (2.28)	*t* = −0.13, *p* = 0.893
	Frequency (%)	Frequency (%)	
Nonsuicidal self-injury (NSSI) at admission	Yes: 28 (29.8) No: 66 (70.2)	Yes: 33 (32.4) No: 69 (67.6)	*χ*^2^*=* 0.15, *p* = 0.698
Type of AN	Restrictive AN: 79 (84.0) Binge-eating/purging AN: 15 (16.0)	Restrictive AN: 90 (88.2) Binge-eating/purging AN: 12 (11.8)	*χ*^2^ = 0.72, *p* = 0.395
Suicidal ideation at admission	Ns Ns	Yes: 16 (15.7) No: 86 (84.3)	

Note. AN = anorexia nervosa; Ns = not detected.

**Table 2:** Descriptive statistics of mentalizing, alexithymia, and psychological difficulties related to eating disorders in two groups.

		Mean	SD	F Test	*p* Value
RFQu	Before-COVID During-COVID	0.88 1.12	0.62 0.64	5.25	0.023
RFQc	Before-COVID During-COVID	0.84 0.80	0.61 0.41	1.88	0.990
TAS–Total	Before-COVID During-COVID	58.7 63.8	14.6 12.3	5.60	0.019
TAS–DIF	Before-COVID During-COVID	21.6 23.8	7.73 6.93	3.53	0.060
TAS–DDF	Before-COVID During-COVID	16.6 18.6	5.34 4.60	6.42	0.012
TAS–EOT	Before-COVID During-COVID	20.7 21.3	6.20 5.28	0.45	0.500
EDI_EDRC	Before-COVID During-COVID	58.8 75.0	26.7 19.8	18.46	<0.001
EDI_IC	Before-COVID During-COVID	58.3 77.5	31.0 23.0	18.51	<0.001
EDI_IPC	Before-COVID During-COVID	57.0 71.7	31.6 26.9	7.54	0.007
EDI_APC	Before-COVID During-COVID	59.6 77.5	30.9 21.6	17.82	<0.001
EDI_OC	Before-COVID During-COVID	53.0 72.7	30.6 25.3	23.88	<0.001
EDI_GPMC	Before-COVID During-COVID	73.7 78.4	34.7 18.4	1.27	0.262

Note. RFQu = uncertainty about mental states; RFQc = certainty about mental states; TAS–Total = alexithymia total score; TAS–DIF = difficulties in identifying feelings; TAS–DDF = difficulties in describing feelings; TAS–EOT = lack of focus on internal emotional experiences; EDI = eating disorder inventory; EDRC = eating disorder risk; IC = ineffectiveness; IPC = interpersonal problems; AP = affective problems; OC = overcontrol; GPMC = global psychological maladjustment.

**Table 3:** Zero-order Spearmans’ correlations between mentalizing, alexithymia, impulsiveness, and EDI composite scales.

	RFQu	TAS–DIF	TAS–DDF	TAS–EOT	BIS–Att.
EDI–EDRC	0.000	0.221	0.3177 **	0.242 *	0.326 *
EDI–IC	0.302 *	0.479 **	0.539 **	0.176	0.487 **
EDI–IPC	0.154	0.413 **	0.669 **	0.105	0.439 **
EDI–APC	0.276 *	0.690 **	0.464 **	0.175	0.562 **
EDI–OC	0.164	0.441 **	0.270 *	0.066	0.338 *
EDI–GPMC	0.344 **	0.709 **	0.605 **	0.158	0.557 **

Note. RFQu = uncertainty about mental states; TAS–DIF = difficulties in identifying feelings; TAS–DDF = difficulties in describing feelings; TAS–EOT = lack of focus on internal emotional experiences; BIS–Att = attentional impulsiveness; EDI = eating disorder inventory; EDRC = eating disorder risk; IC = ineffectiveness; IPC = interpersonal problems; APC = affective problems; OC = overcontrol; GPCMC = global psychological maladjustment; * *p* < 0.05, ** *p* < 0.01.

**Table 4:** Regression model with eating disorder risk (EDI–EDRC) as the dependent variable.

Model *	R	R2	Adjusted R2	R2 Change	df1	df2	Sig. R2 Change
1	0.33	0.11	0.09		1	57	
2	0.41	0.173	0.12	0.06	3	55	0.137

* Model 1 predictor: BIS–Attentional; model 2 predictors: BIS–Attentional + TAS–DDF + TAS–EOT.

**Table 5:** Regression model with global psychological maladjustment (EDI–GPMC) as the dependent variable.

Model *	R	R2	Adjusted R2	R2 Change	df1	df2	Sig. R2 Change
1	0.47	0.22	0.17		3	48	
2	0.66	0.43	0.38	0.21	4	47	<0.001
3	0.69	0.48	0.42	0.05	5	46	0.04
4	0.83	0.69	0.64	0.21	7	44	<0.001

* Model 1 predictors: BMI, NSSI, Age; model 2 predictors: BMI, NSSI, age + BIS–Attentional; model 3 predictors: BMI, NSSI, age + BIS–Attentional + RFQu; model 4 predictors: BMI, NSSI, age + BIS–Attentional + RFQu + TAS–DIF, TAS–DDF.

**Table 6:** Regression model with ineffectiveness (EDI–IC) as the dependent variable.

Model *	R	R2	Adjusted R2	R2 Change	df1	df2	Sig. R2 Change
1	0.36	0.13	0.12		1	55	
2	0.42	0.18	0.14	0.04	2	54	0.09
3	0.57	0.32	0.28	0.15	3	53	0.001
4	0.73	0.53	0.48	0.21	5	51	<0.001

* Model 1 predictor: NSSI; Model 2 predictors: NSSI + RFQu; Model 3 predictors: NSSI + RFQu + BIS–Attentional; Model 4 predictors: NSSI + RFQu + BIS–Attentional + TAS–DIF, TAS–DDF.

**Table 7:** Regression model with interpersonal problems (EDI–IPC) as the dependent variable.

Model *	R	R2	Adjusted R2	R2 Change	df1	df2	Sig. R2 Change
1	0.44	0.19	0.18		1	57	
2	0.71	0.50	0.47	0.31	2	55	<0.001

* Model 1 predictor: BIS–Attentional; model 2 predictors: BIS–Attentional + TAS–DIF, TAS–DDF.

**Table 8:** Regression model with affective problems (EDI–APC) as the dependent variable.

Model *	R	R2	Adjusted R2	R2 Change	df1	df2	Sig. R2 Change
1	0.56	0.31	0.27		3	54	
2	0.58	0.34	0.29	0.03	4	53	0.14
3	0.71	0.50	0.45	0.16	5	52	<0.001
4	0.83	0.70	0.65	0.02	7	50	<0.001

* Model 1 predictor: NSSI; model 2 predictors: NSSI + RFQu; model 3 predictors: NSSI + RFQu + BIS–Attentional; model 4 predictors: NSSI + RFQu + BIS–Attentional + TAS–DIF, TAS–DDF.

**Table 9:** Regression model with overcontrol (EDI–OC) as the dependent variable.

Model *	R	R2	Adjusted R2	R2 Change	df1	df2	Sig. R2 Change
1	0.40	0.16	0.13		2	52	
2	0.53	0.28	0.24	0.12	3	51	0.005
3	0.59	0.34	0.28	0.06	5	49	0.10

* Model 1 predictors: BMI, age; model 2 predictor: BMI, age + BIS-Attentional; model 3 predictor: BMI, age + BIS–Attentional + TAS–DIF, TAS–DDF.

## Data Availability

The data presented in this study are available on request from the corresponding author.
